# Altered Gut Microbiota and Short-Chain Fatty Acids After Vonoprazan-Amoxicillin Dual Therapy for *Helicobacter pylori* Eradication

**DOI:** 10.3389/fcimb.2022.881968

**Published:** 2022-06-02

**Authors:** Yi Hu, Xin Xu, Yao-Bin Ouyang, Cong He, Nian-Shuang Li, Chuan Xie, Chao Peng, Zhen-Hua Zhu, Xu Shu, Yong Xie, Nong-Hua Lu, Yin Zhu

**Affiliations:** ^1^ Department Of Gastroenterology, The First Affiliated Hospital of Nanchang University, Nanchang, China; ^2^ JiangXi Clinical Research Center for Gastroenterology, Nanchang, China

**Keywords:** *Helicobacter pylori*, eradication, vonoprazan, amoxicillin, gut microbiota, short-chain fatty acids

## Abstract

The combination of vonoprazan (VPZ) and amoxicillin (VA therapy) has been shown to achieve acceptable eradication rates for *Helicobacter pylori* (*H. pylori*). Herein, our aim was to explore the short-term effect of VA therapy on the gut microbiota and short-chain fatty acids (SCFAs) using human fecal samples. A total of 119 *H. pylori*-positive patients were randomized into low- or high-dose VA therapy (i.e., amoxicillin 1 g b.i.d. or t.i.d. and VPZ 20 mg b.i.d.) for 7 or 10 days. Thirteen *H. pylori*-negative patients served as controls. Fecal samples were collected from *H. pylori*-positive and *H. pylori*-negative patients. The gut microbiota and SCFAs were analyzed using 16S rRNA gene sequencing and gas chromatography–mass spectrometry, respectively. The gut microbiota in *H. pylori*-positive patients exhibited increased richness, diversity, and better evenness than matched patients. Fifty-three patients studied before and after *H. pylori* eradication were divided into low (L-VA) and high (H-VA) amoxicillin dose groups. The diversity and composition of the gut microbiota among L-VA patients exhibited no differences at the three time points. However, among H-VA patients, diversity was decreased, and the microbial composition was altered immediately after H-VA eradication but was restored by the confirmation time point. The decreased abundance of *Anaerostipes*, *Dialister*, and *Lachnospira* induced by H-VA was associated with altered SCFA levels. VA dual therapy for *H. pylori* eradication has minimal negative effects on gut microbiota and SCFAs.

## Introduction

*Helicobacter pylori (H. pylori)*, a common pathogen that colonizes the stomach, is etiologically associated with diverse gastric and extragastric diseases (gastric cancer, peptic ulcers, chronic gastritis, iron-deficiency anemia, etc.) (Graham, 2015; Amieva and Peek, 2016; Gravina et al., 2020). Studies showing that cure of *H. pylori* infections can reduce the risk of gastric cancer (GC) have resulted in consensus guidelines suggesting the elimination of *H. pylori* for the prevention and control of GC, especially in areas with high incidence of GC (Malfertheiner et al., 2017; El-Serag et al., 2018; Liu et al., 2018; Du et al., 2020; Liou et al., 2020b). Moreover, *H. pylori* infection has also been reported to induce a systematic immunoregulatory effect and to alter the normal acidic gastric environment, leading to alterations of the gastric and gut microbiota (Chen et al., 2021). Our previous animal studies demonstrated that the interactions between *H. pylori*, diet, and the gut microbiota dysregulated host metabolic homeostasis (He et al., 2016; Peng et al., 2021). The call to eliminate *H. pylori* has drawn increasing attention to the perturbation of gut microbiota induced by *H. pylori* eradication.

The extent and severity of perturbations associated with *H. pylori* eradication vary among different regimens in part due to the differences in dose, frequency, and duration of acid inhibitors or the types and duration of antibiotics used (Liou et al., 2020a). For example, Liou et al. (2019) conducted a multicenter, open-label, randomized trial of 1520 patients to evaluate short-term and long-term changes in the gut microbiota induced by triple therapy [amoxicillin, clarithromycin, and a proton pump inhibitor (PPI)] for 14 days, bismuth-containing quadruple therapy (tetracycline, metronidazole, bismuth, and PPI) for 10 days and concomitant therapy (amoxicillin, clarithromycin, metronidazole, and PPI) for 10 days. Alpha diversity was reduced and beta diversity was altered 2 weeks after the end of treatment. Both the alpha diversity and beta diversity were restored more rapidly in those receiving triple therapy vs. bismuth-containing quadruple therapy or concomitant therapy. In addition, the gut microbiota composition was altered at different levels following *H. pylori* eradication during the short-term or interim follow-up, and inconsistent results regarding microbial change were observed during long-term follow-up (Ye et al., 2020). Overall, the current consensus is that *H. pylori* eradication decreases microbial diversity of the gut microbiota but that the composition of the gut microbiota tends to return to a healthy status (He et al., 2019). The long-term effects are unknown but ideally, the preferred therapy would be one with minimal or no detrimental effects on the gut microbiota.

Vonoprazan (VPZ) is a new acid inhibitor that has been available in Japan since 2015. It is widely used for *H. pylori* eradication in Japan due to its strong, fast, and long-lasting ability to inhibit gastric acid (Martinucci et al., 2017; Sugimoto and Yamaoka, 2018). The combination of VPZ (20 mg b.i.d.) and amoxicillin (750 mg b.i.d. or 500 mg t.i.d.), called VA dual therapy, has shown similar efficacy to VPZ-based triple therapy in adults and junior high school students (Furuta et al., 2020; Gotoda et al., 2020; Suzuki et al., 2020). After VPZ triple therapy, the alpha diversity and beta diversity of the gut microbiota were altered at 1 week and 8 weeks compared to baseline. However, no differences were observed when using VA dual therapy, possibly due to the absence of clarithromycin (Horii et al., 2021). One year after treatment, the alpha diversity was increased in both groups in comparison to before eradication (Suzuki et al., 2021). In Japan, the duration of VA dual therapy is 7 days due to limitations by the national medical insurance policy. Currently, the efficacy and safety of VA dual therapy for *H. pylori* eradication in other regions remain unclear, although early reports are promising (Suzuki et al., 2020). The role of VA dual therapy, especially for different durations and doses of amoxicillin and VPZ, on the gut microbiota needs further exploration.

Short-chain fatty acids (SCFAs) are produced in the intestinal tract, primarily depending on the anaerobic fermentation of fiber by intestinal microorganisms (He et al., 2020). Importantly, SCFAs were reported to be involved in regulating energy metabolism and supply, maintaining intestinal barrier integrity, preventing microbial translocation, and decreasing inflammation (Yang et al., 2020). Reductions in SCFAs induced by gut dysbiosis are commonly observed in human metabolic diseases (Morrison and Preston, 2016). *H. pylori* infection has been shown to alter gut SCFA levels in mice. Sodium butyrate, a primary component of SCFAs, inhibits the growth of *H. pylori* and decreases *H. pylori*-induced inflammation, indicating that sodium butyrate might be an efficient metabolite affecting the progression of *H. pylori*-related diseases (Huang et al., 2021). Currently, no clinical trials have been conducted exploring the relationship between SCFAs and *H. pylori* infection or eradication.

In our study, 16S rRNA sequencing and targeted metabolomic profiling (SCFAs) of stool samples were conducted in *H. pylori*-positive and following *H. pylori* eradication and healthy controls. Herein, we aimed to explore the interactions between *H. pylori* infection, the gut microbiota, and SCFAs. Furthermore, alterations in the gut microbiota and SCFAs induced by different doses and durations of VA dual therapies were explored. We also evaluated factors that might influence the gut microbiota and SCFAs. In addition, integrated analysis of gut dysbiosis and SCFAs alterations was performed.

## Materials and Methods

### Study Design and Population

A total of 119 *H. pylori*-positive patients with dyspepsia or health examination were enrolled from the outpatient clinic of The First Affiliated Hospital of Nanchang University and were randomized into four groups: (1) L-VA-10: low dose amoxicillin (1000 mg b.i.d.) and VPZ (20 mg b.i.d.) for 10 days; (2) H-VA-10: high dose amoxicillin (1000 mg t.i.d.) and VPZ (20 mg b.i.d.) for 10 days; (3) L-VA-7: low dose amoxicillin (1000 mg b.i.d.) and VPZ (20 mg b.i.d.) for 7 days; (4) H-VA-7: high dose amoxicillin (1000 mg t.i.d.) and VPZ (20 mg b.i.d.) for 7 days. Fecal samples from 53 *H. pylori*-positive patients with successful eradication were collected, including 17 cases following therapy with L-VA-10, 12 cases following H-LA-10, 13 cases following L-VA-7, and 11 cases following H-LA-7.

The inclusion criteria were (1) age from 18 to 70 years; (2) *H. pylori* infection diagnosed by histology (gastric antrum biopsy was collected and detected for *H. pylori* infection using immunohistochemistry) or ^13^C-urea breath test; and (3) no history of *H. pylori* eradication. The exclusion criteria included (1) allergy to amoxicillin; (2) Zollinger-Ellison syndrome, GC, upper gastrointestinal bleeding, or active peptic ulcer; (3) coexistence of significant concomitant illnesses, including heart disease, renal failure, hepatic disease, previous abdominal surgery, lactation, or pregnancy; (4) use of PPI and antibiotics within the previous one month; and (5) unwillingness to participate in this study.

Thirteen *H. pylori*-negative patients (confirmed by ^13^C-urea breath test) with no history of surgery or other diseases were recruited and defined as healthy controls. Fecal samples were collected at three time points: before eradication therapy, after eradication, and at confirmation of cure of the *H. pylori* infection (confirmation). *H. pylori* eradication was evaluated using the ^13^C-urea breath test 4 weeks after treatment. *H. pylori* status was defined as negative or positive when the delta over baseline was below 4 or above 4 according to the instructions of the manufacturer (HCBT-01, Shenzhen Zhonghe Headway Bio-Sci & Tech Co., Ltd., China). Propensity score matching (PSM) for sex, age, and body mass index between *H. pylori*-positive and *H. pylori*-negative patients (ratio: 2:1) was conducted. Written informed consent was obtained from all patients before enrollment. This study was approved by the Ethics Committee of The First Affiliated Hospital of Nanchang University (2020-024) and registered in the *Chinese Clinical Trial Registry* (ChiCTR2000041477).

### DNA Extraction and 16S rRNA Gene Amplification

Total DNA was extracted using the OMEGA Soil DNA Kit (M5635-02) (Omega Bio-Tek, Norcross, GA, USA) according to the manufacturer’s instructions. The quantity and quality of extracted DNA were measured using a NanoDrop NC2000 spectrophotometer (Thermo Fisher Scientific, Waltham, MA, USA) and agarose gel electrophoresis, respectively. Genomic DNA samples were stored at -20°C prior to further analysis. Polymerase chain reaction amplification of the bacterial 16S rRNA gene V3-V4 region was conducted using the following primers: 338F (5’-ACTCCTACGGGAGGCAGCA-3’) and 806R (5’-GGACTACHVGGGTWTCTAAT-3’). 16S rRNA data were processed as previously described (He et al., 2019). Taxonomy was assigned to amplicon sequence variants (ASVs) using the classify-sklearn naive Bayes taxonomy classifier in the feature-classifier plugin against the Greengenes Database (Desantis et al., 2006).

### Purification and Gas Chromatography–Mass Spectrometry Analysis of Short-Chain Fatty Acids

Samples were thawed on ice, and then 30 mg of each was placed into a 2 mL glass centrifuge tube. Then, 900 μl 0.5% phosphoric acid was added, and samples were shaken for 2 min. Then, the samples were centrifuged at 14,000 × g for 10 min, the supernatant was extracted with 800 μl, and the same amount of ethyl acetate was added for extraction. A total of 600 μl supernatant of the extract was mixed with 500 μM of internal standard (4-methylpentanoic acid) before injection. A mixed standard solution of 7 component methyl esterified fatty acids (Sigma–Aldrich) was used as a reference standard to identify the fatty acids. The quantity of each methyl fatty ester was calculated from the calibration curves of the standards. Eight mixed standard concentration gradients of 0.1 μg/mL, 0.5 μg/mL, 1 μg/mL, 5 μg/mL, 10 μg/mL, 20 μg/mL, 50 μg/mL, and 100 μg/mL were used, where concentration is the total concentration of each component.

The samples were separated on an Agilent DB-WAX capillary column (30 m×0.25 mm ID×0.25 µm) gas chromatography system. The temperature programming was as follows: the initial temperature was 90°C and remained for 3 min. The temperature increased at 10°C/min up to 120°C and then increased at 25°C/min up to 250°C and remained there for 20 min. A QC sample was used for testing and evaluating the stability and repeatability of the system. An Agilent 7890A/5975C gas chromatography-mass spectrometer was used for analysis. The temperatures of the injection port and transmission line were 250°C and 230°C, respectively. The electron bombardment ionization (EI) source, SIM scanning mode, and electron energy were 70 eV. MSD ChemStation software was used to extract the chromatographic peak area and retention time. The content of SCFAs in the sample was calculated by plotting the curve. The quality control samples were processed together with the biological samples. Detected metabolites in pooled samples with a coefficient of variation (CV) less than 30% were denoted as reproducible measurements.

### Bioinformatics and Statistical Analysis

Bioinformatics of the gut microbiome was performed using QIIME2 (Bolyen et al., 2019) with slight modification (https://docs.qiime2.org/2019.4/tutorials/) and R packages (v3.2.0). Briefly, nonsingleton ASVs were aligned and used to construct a phylogeny with fasttree2. Alpha diversity metrics (including Chao1, Shannon, and Pielou’s evenness) were calculated using the ASV table in QIIME2 and are visualized as box plots. ASV-level ranked abundance curves were generated to compare the richness and evenness of ASVs among samples. Beta diversity analysis was conducted to explore the structural variation of microbial communities across samples using Bray–Curtis metrics and was visualized *via* principal coordinate analysis (PCoA). The significance of microbiota structure differences among groups was assessed by PERMANOVA using QIIME2. Linear discriminant analysis effect size (LEfSe) (Segata et al., 2011) was conducted to detect differentially abundant taxa across groups. The linear discriminant analysis threshold was defined as 2, and the Wilcoxon test was used to test the significance of differences in taxa across groups. Microbial functions were predicted using PICRUSt2 in the KEGG (https://www.kegg.jp/) database based on the 16S rRNA sequencing data. Briefly, the ASVs were aligned to reference sequences and placed into reference trees, and the gene family copy numbers of ASVs were then inferred. Gene family abundance per sample was determined, and pathway abundances were inferred. KEGG analysis was conducted using STAMP (Parks et al., 2014). Co-occurrence network analysis was performed using SparCC analysis (abundance>0.1%, r>0.5, Q>0.05). Spearman’s rank-correlation coefficient of the gut microbiota and SCFAs was calculated using Mothur; -1 < rho <0 was considered negatively associated, and 0 < rho <1 was considered positively associated.

Continuous data are presented as the mean ± standard deviation (SD) and were analyzed using one‐way ANOVA. Data with a nonnormal distribution are presented as medians with first and third quartiles and were analyzed using nonparametric statistical tests. SPSS (version 25.0) was used for statistical analysis. *P*<0.05 was considered statistically significant.

### Data Access

All raw sequences were deposited in the NCBI Sequence Read Archive under accession number PRJNA797530.

## Results

### Characteristics of the Study Population

**Figure 1:**
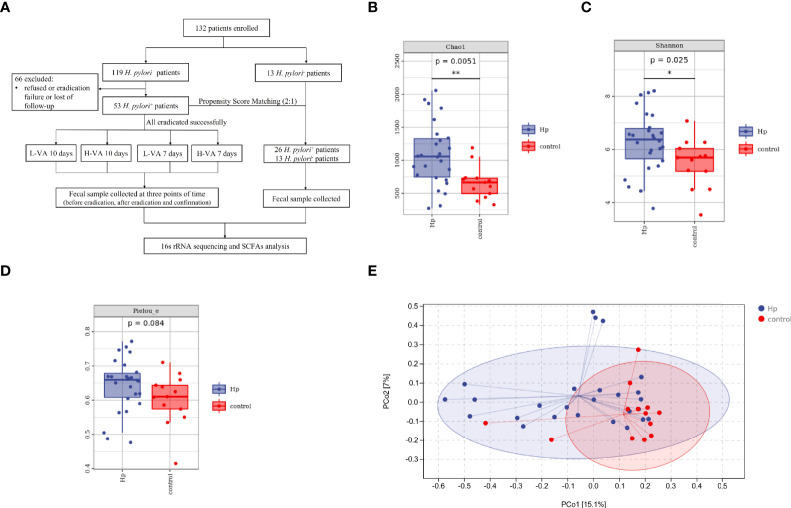
The flowchart of this study and diversity of the gut microbiota between *H. pylori*-positive patients and normal controls. **(A)** Overview of the study design. Bacterial alpha diversity estimated by Chao1 **(B)**, Shannon **(C)**, and Pielou indices **(D)** between *H. pylori*-positive patients and normal controls. **(E)** Bacterial beta diversity estimated by PCoA between *H. pylori*-positive patients and normal controls. L-VA: dual therapy consisting of a low dose of amoxicillin (1000 mg b.i.d.) and VPZ (20 mg b.i.d.); H-VA: dual therapy consisting of a high dose of amoxicillin (1000 mg t.i.d.) and VPZ (20 mg b.i.d.); SCFAs, short-chain fatty acids; Hp, *H. pylori*-positive; Control, *H. pylori*-negative; PCoA, principal coordinate analysis. *P < 0.05, **P < 0.01.

From January 9, 2021, to October 1, 2021, 132 patients were enrolled in this study, including 119 *H. pylori*-positive and 13 *H. pylori*-negative patients. Fecal samples from 66 *H. pylori*-positive patients were not collected due to refusal, eradication failure, or loss to follow-up. The final study population consisted of 53 *H. pylori*-positive patients whose *H. pylori* infection was successfully eradicated with VA dual therapy, and 13 *H. pylori*-negative patients were further included to analyze alteration in the gut microbiota and SCFAs. Twenty-six of the 53 *H. pylori*-positive patients were screened using the PSM (ratio 2:1) method to balance the baseline characteristics between the *H. pylori*-positive and *H. pylori*-negative groups (**Figure 1A**). The gender, sex, and body mass index between the two groups exhibited no differences (**Table S1**). The L-VA-10 and L-VA-7 groups were combined as the L-VA group (daily dose of amoxicillin ≤ 2 g and total dosedose ≤ 20 g), and the H-VA-10 and H-VA-7 groups were combined as the H-VA group (daily dose of amoxicillin>2 g and total dose>20 g). Detailed sample information of the L-VA and H-VA groups at three time points (before eradication therapy, after eradication, and confirmation for *H. pylori*) is shown in **Table S2**.

### *H. pylori* Infection, The Gut Microbiota, and SCFAs

#### Diversity and Compositional Analysis

**Figure 2:**
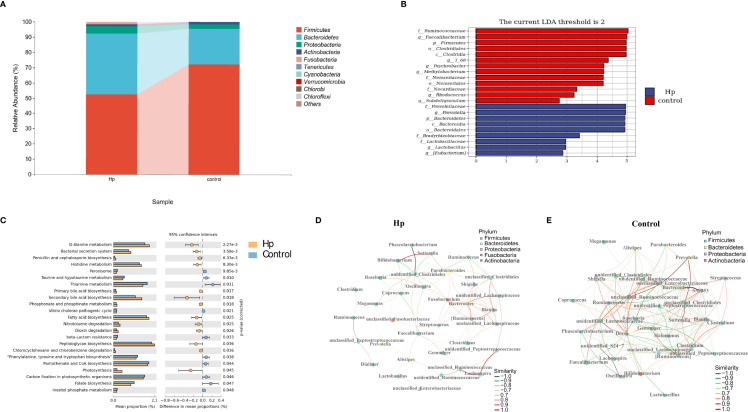
The composition and predicted functional differences between *H. pylori*-positive patients and normal controls. **(A)** The composition of the gut microbiota between *H. pylori*-positive patients and normal controls at the phylum level (the abundance of the top 10 is shown). **(B)** Differences in specific bacterial taxa between *H. pylori*-positive and normal controls by LEfSe. **(C)** Significant signaling pathways associated with *H.pylori* infection according to KEGG pathway analysis. Ecological co-occurrence network between *H. pylori*-positive **(D)**, and normal controls **(E)** Hp: *H.pylori*-positive; Control: *H. pylori*-negative.

We first compared the alpha and beta diversity between the 26 *H. pylori*-positive patients and the 13 *H. pylori*-negative patients. The Chao1 index indicated that the gut microbiota in the *H. pylori*-positive group exhibited increased richness compared to that in the *H. pylori*-negative group (*P*<0.05, **Figure 1B**). Higher diversity was also observed in *H. pylori*-positive patients than in *H. pylori*-negative patients, as revealed by the Shannon index (*P*<0.05, **Figure 1C**). The Pielou index revealed that *H. pylori*-positive patients displayed better evenness than negative patients, although the difference was not significant (*P*=0.084, **Figure 1D**). PCoA was performed to evaluate alterations in community composition, and distinct clustering was found between *H. pylori*-positive and *H. pylori*-negative groups as measured by Bray–Curtis metrics (*P*<0.05, **Figure 1E**). Differential bacterial compositions were observed between the two groups. At the phylum level, the relative abundance of *Firmicutes* and *Actinobacteria* was increased, while the relative abundance of *Bacteroidetes*, *Proteobacteria* and *Fusobacteria* was reduced in the *H. pylori*-negative group compared to the *H. pylori*-positive group (**Figure 2A**). Significant differences in taxa were observed between the two groups using LEfSe (**Figure 2B**).

#### Functional Analysis

We also explored the predictive functional profiling using 16S rRNA sequencing data. Twenty-three signaling pathways were significantly different between the *H. pylori*-positive and *H. pylori*-negative groups (*P*<0.05, **Figure 2C**), primarily focusing on metabolic signaling pathways (D-alanine metabolism, histidine metabolism, taurine and hypotaurine metabolism, etc.) and biosynthesis signaling pathways (penicillin and cephalosporin biosynthesis, primary and secondary bile acid biosynthesis, fatty acid biosynthesis, etc.). Network topology analysis showed that the closeness centrality, number of edges, average nearest neighbor degree, and degree centralization were higher in the *H. pylori*-negative group than in the *H. pylori*-positive group (**Figures 2D, E**; **Table S3**).

#### SCFA Analysis

**Figure 3:**
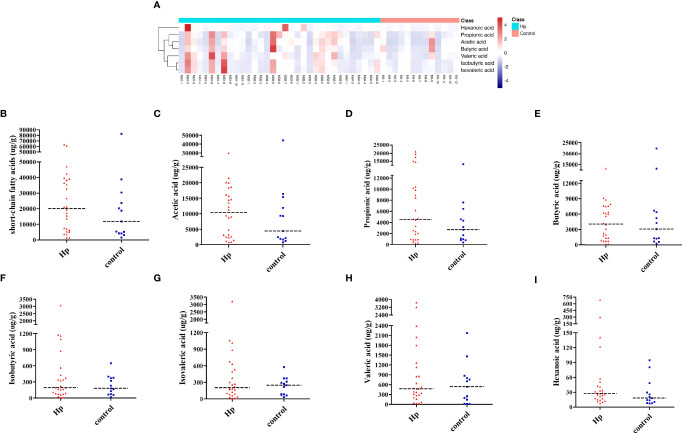
Analysis of SCFAs in *H. pylori*-positive patients and normal controls. **(A)** Heatmap of SCFA differences across different samples in *H. pylori*-positive and normal control groups. The quantification of total SCFAs **(B)**, acetic acid **(C)**, propionic acid **(D)**, butyric acid **(E)**, isobutyric acid **(F)**, isovaleric acid **(G)**, valeric acid **(H)**, and hexanoic acid **(I)** in the *H. pylori*-positive patients and normal control groups. Hp: *H. pylori*-positive; Control: *H. pylori*-negative.

SCFAs included acetic acid, propionic acid, butyric acid, isobutyric acid, isovaleric acid, valeric acid, and hexanoic acid, which were measured in the *H. pylori*-positive and *H. pylori*-negative groups. As shown in **Figure 3A**, the *H. pylori*-positive group tended to have a slightly higher abundance of SCFAs than the *H. pylori*-negative group, and differences in the subjects within each group were also observed. Among SCFAs, acetic acid, propionic acid, and butyric acid were the most abundant (>90%). Levels of total SCFAs, acetic acid and propionic acid were higher in the *H. pylori*-positive group than in the *H. pylori*-negative group, although the differences were not statistically significant (**Figures 3B–D**; **Table S4**). Levels of butyric acid, isobutyric acid, isovaleric acid, valeric acid, and hexanoic acid were not different between the two groups (*P*>0.05, **Figures 3E–I**; **Table S4**).

### *H. pylori* Eradication, VA Dual Therapy, Gut Microbiota, and SCFAs

#### Diversity and Compositional Analysis

**Figure 4:**
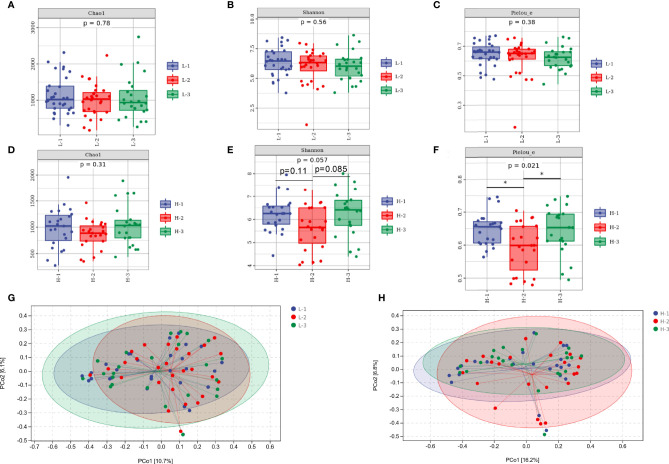
The diversity of the gut microbiota between the L-VA and H-VA therapy groups. Bacterial alpha diversity estimated by Chao1 **(A)**, Shannon **(B)**, and Pielou indices **(C)** at the three time points of L-VA therapy. The Chao1 **(D)**, Shannon **(E)**, and Pielou indices **(F)** at the three time points of H-VA therapy. Bacterial beta diversity estimated by PCoA at three time points of L-VA **(G)** and H-VA **(H)** therapy. L-VA: dual therapy consisting of a low dose of amoxicillin (1000 mg b.i.d.) and VPZ (20 mg b.i.d.); H-VA: dual therapy consisting of a high dose of amoxicillin (1000 mg t.i.d.) and VPZ (20 mg b.i.d.); L-1: the time point before eradication in the L-VA group; L-2: the time point after eradication in the L-VA group; L-3: the time point of confirmation in the L-VA group; H-1: the time point before eradication in the H-VA group; H-2: the time point after eradication in the H-VA group; H-3: the time point of confirmation in the H-VA group; * *P*<0.05.

As shown in **Figures 4A–C**, richness, diversity, and evenness were not altered by L-VA therapy, as revealed by the Chao1, Shannon, and Pielou indices. The richness was slightly reduced after eradication and increased at confirmation when receiving H-VA therapy, although the differences were not statistically significant (**Figure 4D**). The Shannon and Pielou indices showed that the diversity and evenness were decreased after receiving H-VA therapy and increased at confirmation (Shannon index: before eradication vs. after eradication *P*=0.11, after eradication vs. confirmation *P*=0.085; Pielou index: before eradication vs. after eradication *P*<0.05, after eradication vs. confirmation *P*<0.05; **Figures 4E, F**). No distinct clustering was observed at the three time points of L-VA therapy (*P*>0.05, **Figure 4G**). However, different community compositions were observed among the time points before eradication and after eradication when subjects received H-VA therapy (*P*<0.05, **Figure 4H**), but no distinct clustering was found when comparing the time points before eradication and confirmation (*P*>0.05, **Figure 4H**).

**Figure 5:**
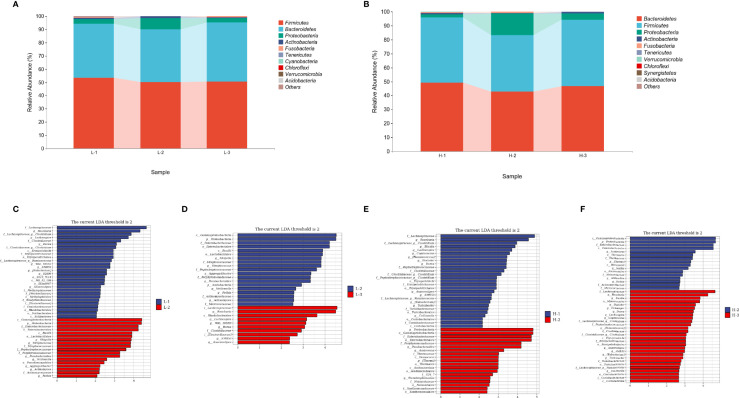
Composition differences at the three time points in L-VA and H-VA therapy. The composition of the gut microbiota between L-VA **(A)** and H-VA **(B)** therapy at the phylum level (the abundance of Top 10 are shown). Differences in specific bacterial taxa when comparing L-1 and L-2 **(C)**, L-2 and L-3 **(D)**, H-1 and H-2 **(E)**, and H-2 and H-3 **(F)** by LEfSe. L-1: the time point before eradication in the L-VA group; L-2: the time point after eradication in the L-VA group; L-3: the time point of confirmation in the L-VA group; H-1: the time point before eradication in the H-VA group; H-2: the time point after eradication in the H-VA group; H-3: the time point of confirmation in the H-VA group.

At the phylum level, the abundances of *Firmicutes* and *Bacteroidetes*, accounting for >90% abundance of gut microbiota, were similar at the three time points of L-VA therapy. A slightly increased abundance of *Proteobacteria* and *Actinobacteria* and a slightly decreased abundance of *Fusobacteria* were observed after L-VA eradication in comparison to the time points before eradication and confirmation (**Figure 5A**). Notably, among the H-VA group, a distinct decrease in the abundance of *Firmicutes*, *Bacteroidetes* and *Actinobacteria* and an increase in the abundance of *Proteobacteria* were observed after eradication compared to before eradication or confirmation (**Figure 5B**). LEfSe for L-VA therapy revealed differences in taxa after eradication compared to before eradication or confirmation (**Figures 5C, D**). Interestingly, *Rosebubria* and *Anaerostipes* (SCFA producers) were decreased after eradication and increased at confirmation. As shown in **Figures 5E, F**, distinct differences in taxa were observed after H-VA eradication compared to before eradication and confirmation. A similar trend of *Rosebubria*, *Eubacterium, Blautia*, *Anaerostipes, Dialister*, and *Lachnospira* abundance among the three time points was also observed in the H-VA group.

#### Functional Analysis

The KEGG pathway analysis revealed that 25 and 19 signaling pathways were enriched when comparing the time point after eradication to before eradication and confirmation among the L-VA group **(** *P*<0.05, **Supplementary Figures S1A, B**). Compared to L-VA therapy, more significant signaling pathways were enriched among H-VA therapy (after eradication vs. before eradication: 59 signaling pathways; after eradication vs. confirmation: 32 signaling pathways) (*P*<0.05, **Supplementary Figures 1D, E**). Only 3 and 1 signaling pathways were enriched from before eradication to confirmation between the L-VA and H-LA groups (*P*<0.05, **Supplementary Figures 1C, F**). Network topology analysis of L-VA therapy showed that the indices of average nearest neighbor degree, closeness centrality, transitivity, and edge number were decreased after eradication and restored at the time point of confirmation (**Supplementary Figures 2A, C**, **Table S5**). Among the H-VA group, the indices of average nearest neighbor degree and closeness centrality were decreased after eradication and increased at the time point, confirmation. Moreover, the edge number index was highest at the time point (**Supplementary Figures 2D–F**; **Table S6**).

#### SCFA Analysis

**Figure 6:**
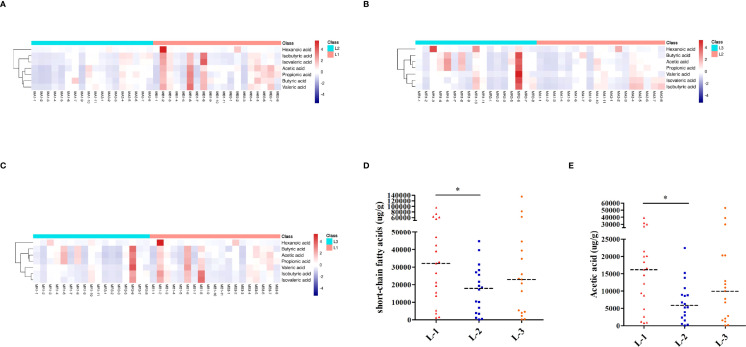
SCFA analysis of L-VA therapy. Heatmap of SCFA differences across different samples at the three time points of L-VA therapy (**A** for L-1 and L-2, **B** for L-2 and L-3, **C** for L-1 and L-3). The quantification of total SCFAs **(D)** and acetic acid **(E)** at the three time points of L-VA therapy. Red represents a positive association, and blue represents a negative association. L-1: the time point before eradication in the L-VA group; L-2: the time point after eradication in the L-VA group; L-3: the time point of confirmation in the L-VA group; * *P*<0.05.

As shown in **Figures 6A–C**, the heatmap of SCFAs among different samples showed that SCFAs after eradication by L-VA therapy were different from those before eradication and confirmation. We also observed SCFA differences across samples within each group. Levels of total SCFAs and acetic acid were decreased after eradication and increased at confirmation (**Figures 6D, E**; **Table S7**). Levels of propionic acid, butyric acid, isobutyric acid, isovaleric acid, valeric acid, and hexanoic acid exhibited no significant differences at any of the three time points in L-VA therapy (**Supplementary Figures 3A–F**, **Table S7**).

**Figure 7:**
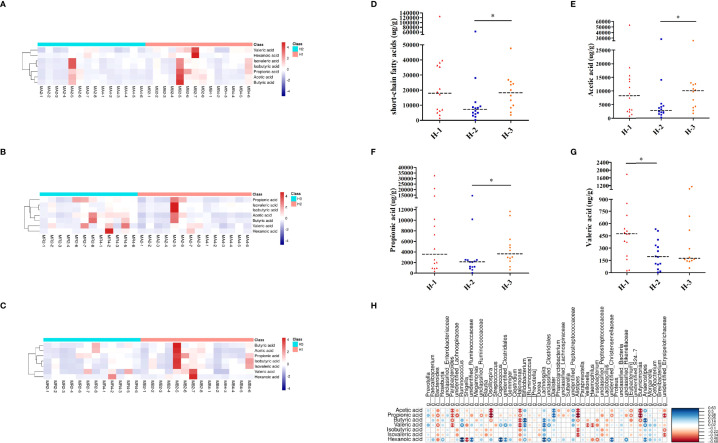
SCFA analysis of H-VA therapy. Heatmap of SCFA differences across different samples at the three time points of H-VA therapy (**A** for H-1 and H-2, **B** for H-2 and H-3, **C** for H-1 and H-3), red represents a positive association, and blue represents a negative association. The quantification of total SCFAs **(D)**, acetic acid **(E)**, propionic acid **(F)**, and valeric acid **(G)** at the three time points of H-VA therapy. **(H)** Integrated analysis of SCFA alterations associated with gut microbiota among the H-VA group, blue represents a positive association, and red represents a negative association. H-1: the time point before eradication in the H-VA group; H-2: the time point after eradication in the H-VA group; H-3: the time point of confirmation in the H-VA group; * *P*<0.05.

As shown in **Figures 7A–C**, the heatmap of SCFAs among different samples of H-VA therapy showed that SCFAs after eradication were different from those before eradication and confirmation. SCFA differences across samples within each group were also observed. Levels of total SCFAs, acetic acid and propionic acid were decreased after eradication and restored at confirmation (**Figures 7D–F**; **Table S8**). Levels of valeric acid were decreased after eradication **(** **Figure 7G**; **Table S8**). No significant differences were observed for levels of butyric acid, isobutyric acid, isovaleric acid, or hexanoic acid at any of the three time points in H-VA therapy (**Supplementary Figures 4A–D**; **Table S8**). The integrated analysis of gut microbiota and SCFA alterations indicated that *Anaerostipes, Dialister, and Lachnospira* were positively associated with SCFAs, and their expression was decreased after eradication and increased, confirmation (**Figures 5E, F**,**7H**).

## Discussion

To our knowledge, this is the first randomized clinical trial to evaluate the short-term effect of different VA therapies on the gut microbiota and SCFAs. Moreover, we addressed the relationship between *H. pylori* infection and alterations in the gut microbiota and SCFAs. Previous studies (Heimesaat et al., 2014; Ge et al., 2018) established an *H. pylori*-infected Mongolian gerbil model for 14 months and a C57BL/6 mouse model for 4 months to explore the influence of *H. pylori* infection on the gut microbiota. Both short-term and long-term *H. pylori* infection altered the composition of the gut microbiota. The majority of clinical trials in adults (Frost et al., 2019; Wang et al., 2019; Iino et al., 2020) and our previous study (He et al., 2019) have identified the distinct clustering of gut microbiota between *H. pylori-*positive subjects and normal controls using the next-generation sequencing method, and a higher diversity of gut microbiota was observed in the *H. pylori-*positive group. In our study, PSM was used to match *H. pylori*-positive subjects to normal controls. The alpha diversity was higher in the *H. pylori-*positive group than in the *H. pylori-*negative group. Distinct clustering and different bacterial compositions were observed between the two groups. In addition, *H. pylori* infection might affect the interactions of gut microbiota.

Huang et al. (2021) first explored the relationship between *H. pylori* infection and SCFAs using an *H. pylori*-infected C57BL/6 mouse model. The results showed that *H. pylori* infection decreased the levels of acetic acid, propionic acid, butyric acid, isobutyric acid, isovaleric acid, valeric acid, and hexanoic acid. The number of mice included in each group was limited (n=6). In our clinical trial, we included 26 *H. pylori*-positive and 13 *H. pylori*-negative subjects for SCFA analysis, and slightly higher abundances of total SCFAs, acetic acid, and propionic acid were observed in *H. pylori-*positive patients than in *H. pylori*-negative patients. However, considering the sample size and the differences within groups, this difference was not statistically significant. Moreover, we found no association of gut microbiota alterations with SCFA levels. The relationship between *H. pylori* infection and SCFAs needs to be further explored.

Antibiotics comprise the primary regimens for eradicating *H. pylori* but are a double-edged sword, as they are involved in disrupting the homeostasis of the gut microbiota (Ianiro et al., 2016). Multiple studies (Hsu et al., 2018; Hsu et al., 2019; Martin-Nunez et al., 2019) and our previous study (He et al., 2019) reported that significant perturbations were induced in the gut microbiota immediately following *H. pylori* eradication and that the gut microbiota alterations were not completely restored short-term. Triple or quadruple therapy, concomitant therapy, and hybrid therapy have been used in most studies and contain many broad-spectrum antimicrobials, including bismuth, tetracycline, clarithromycin, and metronidazole. Not surprisingly, the extent and severity of perturbation induced by *H. pylori* eradication vary among different regimens. Compared to quadruple therapy, the gut microbiota was restored more rapidly following triple therapy during long-term follow-up (Liou et al., 2019), which was primarily attributed to the fact that triple therapy used one less drug. VA dual therapy contains only one antibiotic, amoxicillin, which has a limited spectrum but can achieve a similar efficacy for eradicating *H. pylori* in high clarithromycin resistance regions, especially with VPZ (Suzuki et al., 2020), and has less impact on the gut microbiota (Horii et al., 2021). The diversity of the gut microbiota was not influenced by VA dual therapy (VPZ 20 mg b.i.d. and amoxicillin 750 mg b.i.d.) administered for 7 days. VA dual therapy is only now being introduced in other regions, and it has not yet been optimized to reliably achieve high cure rates. It remains unclear what the impact of increasing the dose and/or duration of amoxicillin will have on the gut microbiota. As such, our study first explored the impact of different VA therapies on the gut microbiota and analyzed the role of amoxicillin dose in the alteration of gut microbiota.

We divided VA dual therapies with different durations and doses of amoxicillin. L-VA therapy exerted no impacts on the richness, diversity, or evenness of gut microbiota immediately after eradication. Moreover, no distinct clustering was observed before eradication, after eradication, or at confirmation. However, the diversity was reduced, and perturbations of gut microbiota were induced immediately after H-VA dual therapy. This alteration tended to be restored by confirmation. Ye et al. (Ye et al., 2020) recently conducted a meta-analysis of the gut microbiota composition in response to *H. pylori* eradication. At the phylum level, a decreased abundance of *Actinobacteria* and an increased abundance of *Proteobacteria* were observed during short-term follow-up. In addition, *Firmicutes* and *Bacteroidetes* tended to decrease immediately after eradication (Chen et al., 2021). The same trend of gut microbiota alterations at the phylum level were observed in H-VA therapy. Functional analysis demonstrated that more signaling pathways were enriched in H-VA therapy than in L-VA therapy, which could be explained by the distinct alterations of the gut microbiota induced by H-VA therapy. The bacterial interactions were influenced after eradication with L-VA and H-VA therapy and recovered confirmation, which was confirmed by network topology analysis. The diversity, composition, and function of the gut microbiota were more strongly influenced by H-VA therapy, which quickly recovered during a short period.

Currently, no clinical trials have addressed the impact of *H. pylori* eradication on SCFAs. We first demonstrated that *H. pylori* eradication leads to a decreased level of SCFAs, which was related to gut dysbiosis. The impact was more distinct with H-VA therapy. Most SCFA alterations recovered by 4 weeks after eradication. *Anaerostipes* was shown to have the capability to produce butyrate and propionate, which are associated with host health (Bui et al., 2021). *Dialister* and *Lachnospira* are also reported to be producers of SCFAs (Koh et al., 2016; Fitzgerald et al., 2021). Our integrated analysis of the gut microbiota and SCFA alterations revealed that the decreased abundance of *Anaerostipes, Dialister, and Lachnospira* induced by H-VA therapy might be involved in the alteration of SCFA levels. Our results provide evidence regarding the relationship between *H. pylori* eradication, the gut microbiota, and SCFA alterations.

There are some limitations to this study. First, a total of 53 *H. pylori*-positive subjects and 13 normal controls from one center in China were included, which cannot completely reflect the impact of VA dual therapy on gut microbiota and SCFAs in other regions or population groups. Multicenter clinical trials with a larger number of subjects from additional regions are warranted. Second, mucosa-associated or gastric biopsies were not obtained because endoscopy was not conducted in a majority of subjects. As such, we could not analyze the influence of VA dual therapy on the local diversity of gastric or gut microbiota. Third, because VPZ has a strong ability to inhibit gastric acid secretion, further dose-related studies regarding its effects on the gut microbiota are warranted. The dose of VPZ used in this study was 20 mg twice daily. As such, the influence of different VPZ doses on the gut microbiota could not be analyzed. Fourth, the long-term impact of VA dual therapies on the gut microbiota was not analyzed. We found that gut dysbiosis was restored 4 weeks after *H. pylori* eradication.

In conclusion, our study demonstrated that *H. pylori* infection induces alterations in the gut microbiota. L-VA therapy exerted no or little influence on the diversity and composition of the gut microbiota. H-VA therapy had a greater impact on the gut microbiota than L-VA therapy. Gut dysbiosis occurred immediately after eradication but was quickly restored by 4 weeks post-therapy. Decreased levels of SCFAs were observed in the L-VA and H-VA groups and increased 4 weeks after eradication. Linkages between *H. pylori* eradication, gut dysbiosis, and SCFA alterations were identified. These results indicate that VA dual therapy induces minimal effects on the gut microbiota and SCFAs, supporting its short-term use and safety.

## Data Availability Statement

The datasets presented in this study can be found in online repositories. The names of the repository/repositories and accession number(s) can be found in the article/**Supplementary Material**.

## Ethics Statement

The studies involving human participants were reviewed and approved by The Ethics Committee of The First Affiliated Hospital of Nanchang University (2020-024). The patients/participants provided their written informed consent to participate in this study.

## Author Contributions

YH and XX collected the fecal samples, recorded the basic information, and analyzed the sequencing data. Y-BO, CH, N-SL, CX, and CP provided suggestions for the study design and participated in analyzing the sequencing data. Z-HZ, XS, and YX provided suggestions for the study design and revised the manuscript. YH, N-HL, and YZ designed the study and wrote and edited the manuscript. All authors contributed to the article and approved the submitted version.

## Funding

This study was supported by the National Natural Science Foundation of China (NO. 82000531 and 82170580); the Project for Academic and Technical Leaders of Major Disciplines in Jiangxi Province (NO. 20194BCJ22016); the Key Research and Development Program of Jiangxi Province (NO. 20212BBG73018); the Youth Project of the Jiangxi Natural Science Foundation (NO. 20202BABL216006); the Key Fund of the Jiangxi Education Department (NO. GJJ190007); the Scientific Research of Health Commission of Jiangxi Province (NO.20213019); Scientific Research of Traditional Chinese Medicine of Jiangxi Province (NO.2020A0047); and the Young Teachers’ Scientific Research and Cultivation Fund of the Medical Department of Nanchang University (PY201919).

## Conflict of Interest

The authors declare that the research was conducted in the absence of any commercial or financial relationships that could be construed as a potential conflict of interest.

## Publisher’s Note

All claims expressed in this article are solely those of the authors and do not necessarily represent those of their affiliated organizations, or those of the publisher, the editors and the reviewers. Any product that may be evaluated in this article, or claim that may be made by its manufacturer, is not guaranteed or endorsed by the publisher.

## Supplementary Material

Supplementary Figure 1 KEGG pathway analysis of L-VA and H-LA therapy. Significant signaling pathways enriched by comparing L-1 and L-2 **(A)**, L-2 and L-3 **(B)**, L-1 and L-3 **(C)**, H-1 and H-2 **(D)**, H-2 and H-3 **(E)**, H-1 and H-3 **(F)** according to KEGG pathway analysis. L-1: before eradication in the L-VA group; L-2: after eradication in the L-VA group; L-3: confirmation in the L-VA group; H-1: before eradication in the H-VA group; H-2: after eradication in the H-VA group; H-3: confirmation in the H-VA group.

Supplementary Figure 2 Ecological co-occurrence network analysis of L-VA and H-LA therapies. The network analysis before eradication (**A** for L-VA therapy and D for H-VA therapy), after eradication (**B** for L-VA therapy and E for H-VA therapy) and confirmation (**C** for L-VA therapy and F for H-VA therapy). L-VA: Dual therapy consisting of a low dose of amoxicillin (1000 mg b.i.d.) and VPZ (20 mg b.i.d.); H-VA: dual therapy consisting of a high dose of amoxicillin (1000 mg t.i.d.) and VPZ (20 mg b.i.d.).

Supplementary Figure 3 SCFAs analysis of L-VA therapy. The quantification of propionic acid **(A)**, butyric acid **(B)**, isobutyric acid **(C)**, isovaleric acid **(D)**, valeric acid **(E)**, and hexanoic acid **(F)** at the three time points of L-VA therapy. L-1: before eradication in the L-VA group; L-2: after eradication in the L-VA group; L-3: confirmation in the L-VA group.

Supplementary Figure 4 SCFAs analysis of H-VA therapy. The quantification of butyric acid **(A)**, isobutyric acid **(B)**, isovaleric acid **(C)**, and hexanoic acid **(D)** at the three time points of H-VA therapy. H-1: before eradication in the H-VA group; H-2: after eradication in the H-VA group; H-3: confirmation in the H-VA group.
