# Supplementary figures and images for: Altered Gut Microbiota and Short-Chain Fatty Acids After Vonoprazan-Amoxicillin Dual Therapy for Helicobacter pylori Eradication
Source: Front Cell Infect Microbiol. 2022 Jun 2;12:881968. doi: 10.3389/fcimb.2022.881968 (PMC9201212; doi:10.3389/fcimb.2022.881968)

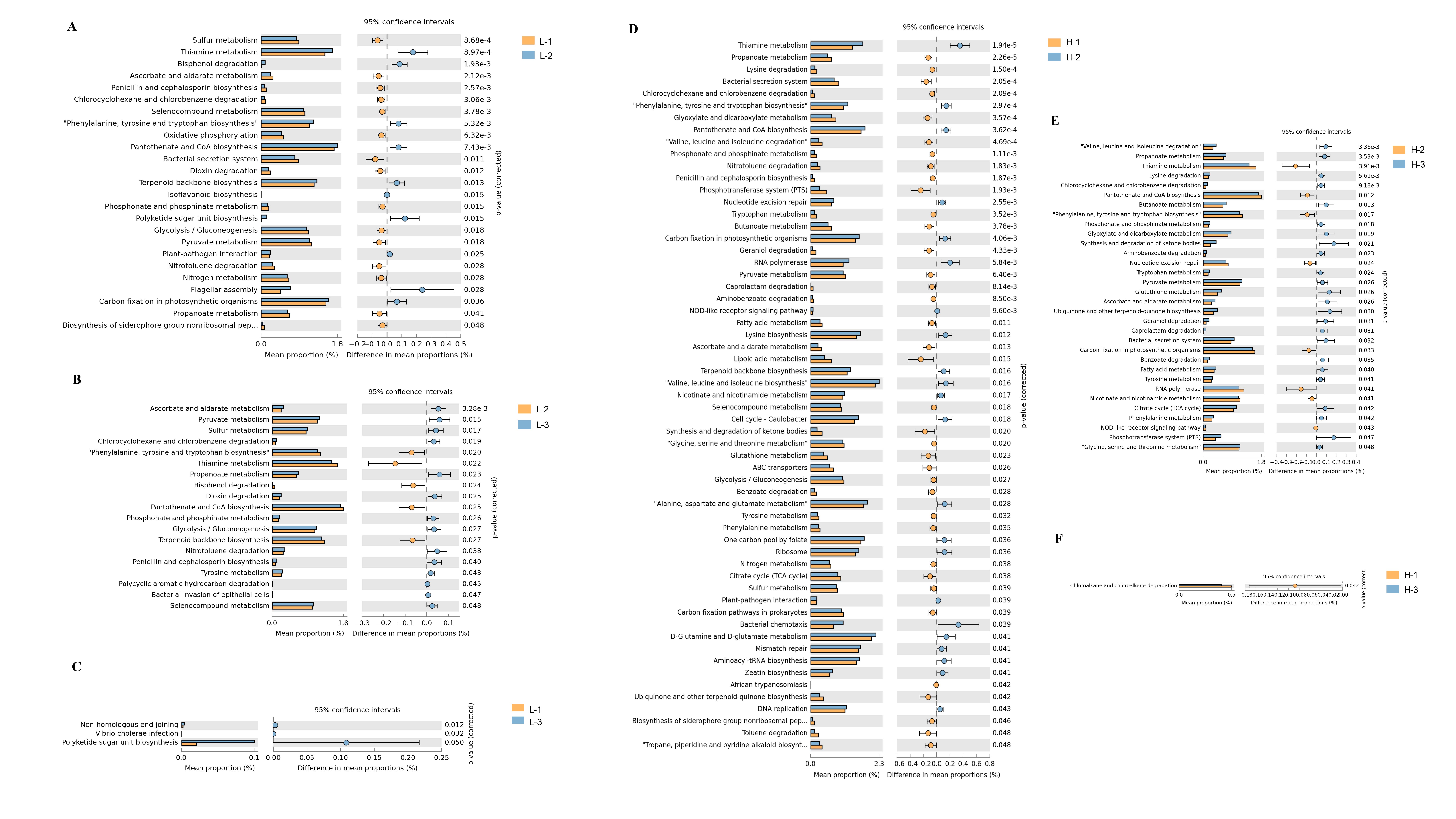

Supplement: Supplementary Figure 1 — KEGG pathway analysis of L-VA and H-LA therapy. Significant signaling pathways enriched by comparing L-1 and L-2 (A), L-2 and L-3 (B), L-1 and L-3 (C), H-1 and H-2 (D), H-2 and H-3 (E), H-1 and H-3 (F) according to KEGG pathway analysis. L-1: before eradication in the L-VA group; L-2: after eradication in the L-VA group; L-3: confirmation in the L-VA group; H-1: before eradication in the H-VA group; H-2: after eradication in the H-VA group; H-3: confirmation in the H-VA group. [file Image_1.jpeg]

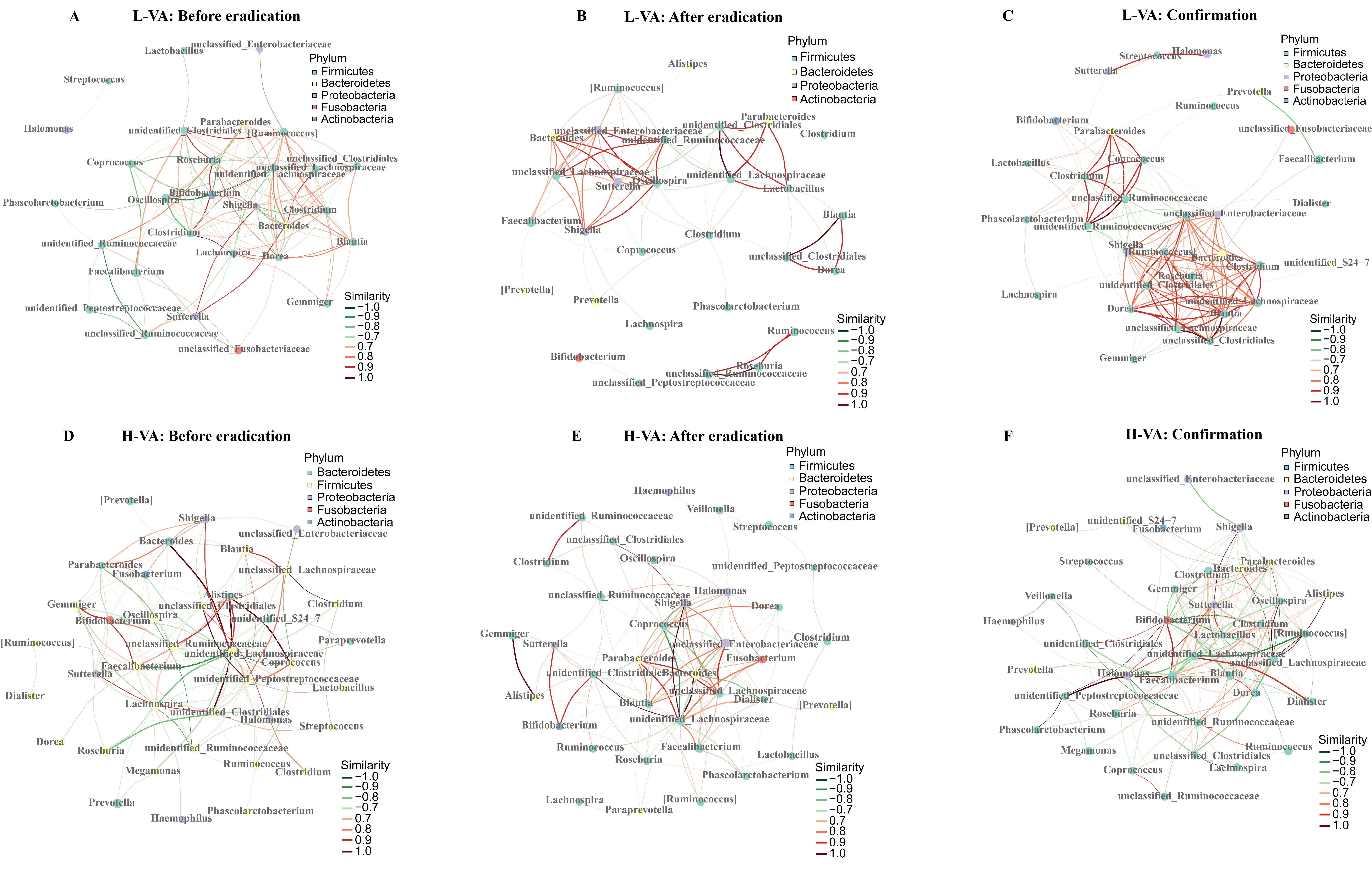

Supplement: Supplementary Figure 2 — Ecological co-occurrence network analysis of L-VA and H-LA therapies. The network analysis before eradication (A for L-VA therapy and D for H-VA therapy), after eradication (B for L-VA therapy and E for H-VA therapy) and confirmation (C for L-VA therapy and F for H-VA therapy). L-VA: Dual therapy consisting of a low dose of amoxicillin (1000 mg b.i.d.) and VPZ (20 mg b.i.d.); H-VA: dual therapy consisting of a high dose of amoxicillin (1000 mg t.i.d.) and VPZ (20 mg b.i.d.). [file Image_2.jpeg]

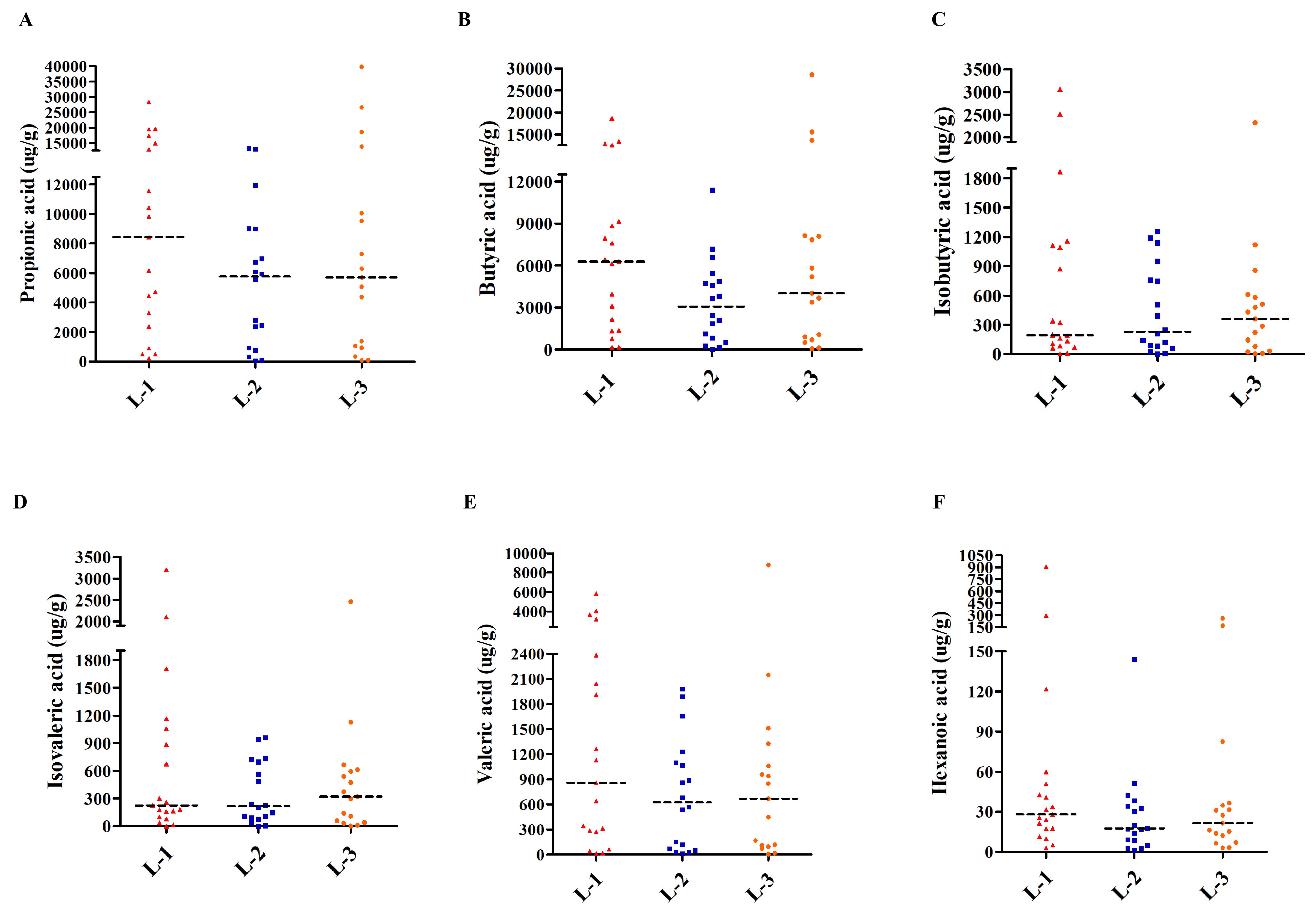

Supplement: Supplementary Figure 3 — SCFAs analysis of L-VA therapy. The quantification of propionic acid (A), butyric acid (B), isobutyric acid (C), isovaleric acid (D), valeric acid (E), and hexanoic acid (F) at the three time points of L-VA therapy. L-1: before eradication in the L-VA group; L-2: after eradication in the L-VA group; L-3: confirmation in the L-VA group. [file Image_3.jpeg]

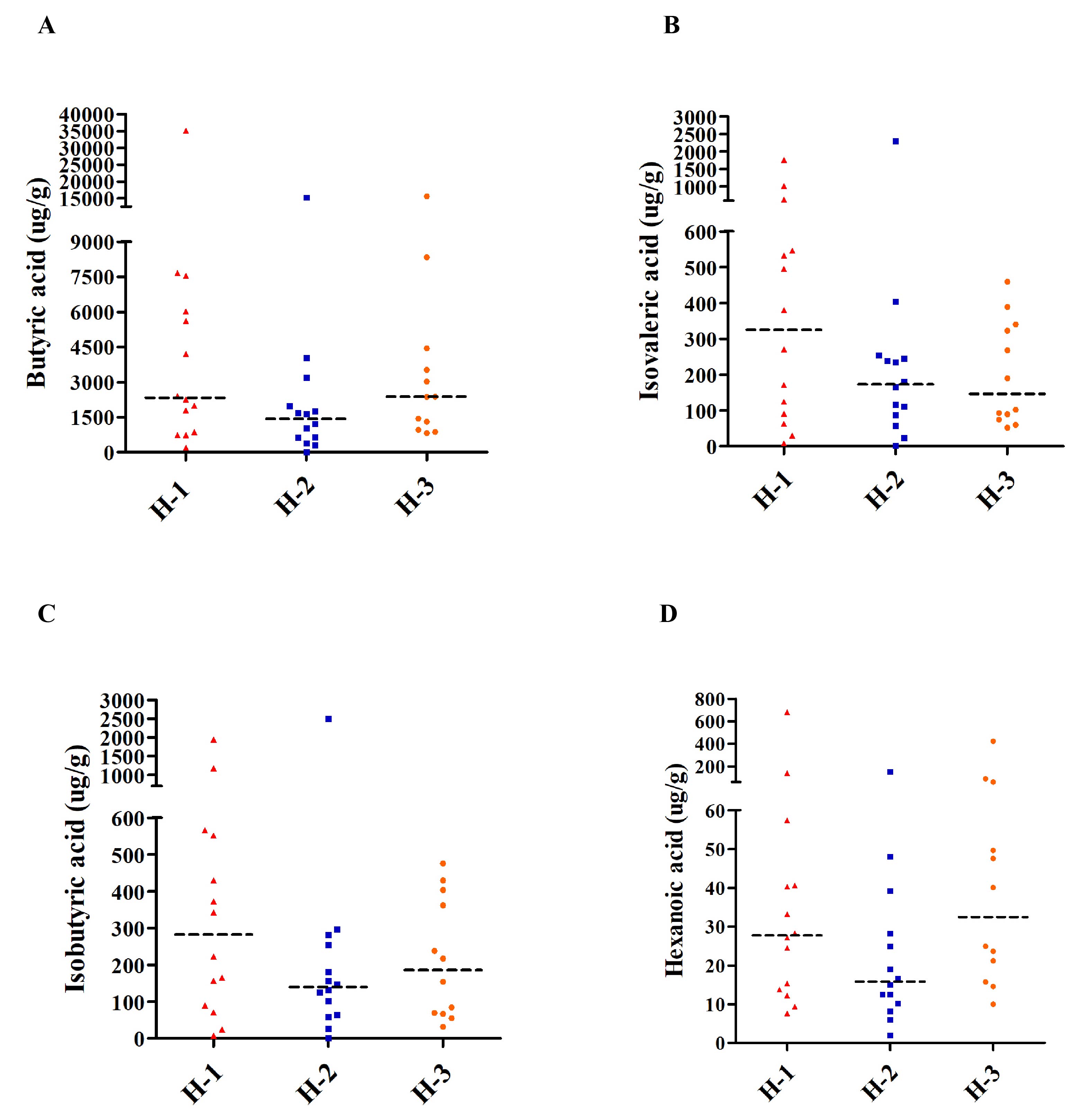

Supplement: Supplementary Figure 4 — SCFAs analysis of H-VA therapy. The quantification of butyric acid (A), isobutyric acid (B), isovaleric acid (C), and hexanoic acid (D) at the three time points of H-VA therapy. H-1: before eradication in the H-VA group; H-2: after eradication in the H-VA group; H-3: confirmation in the H-VA group. [file Image_4.jpeg]
