# Supplementary material for: Altered Gut Microbiota and Short-Chain Fatty Acids After Vonoprazan-Amoxicillin Dual Therapy for Helicobacter pylori Eradication
Source: Front Cell Infect Microbiol. 2022 Jun 2;12:881968. doi: 10.3389/fcimb.2022.881968 (PMC9201212; doi:10.3389/fcimb.2022.881968)
Supplement: Supplementary file 5 [file Table_1.docx]

**Table S1 Clinicopathological characteristics of patients in the *H. pylori*-positive and -negative groups**

|  | ***H. pylori-positive***  **(n=26)** | ***H. pylori*-negative**  **(n=13)** | ***P* value** |
| --- | --- | --- | --- |
| Gender (Male/Female)  Age  Body Mass Index | 10/16  32.27±9.46  21.86±2.73 | 4/9  29.85±12.58  21.10±2.41 | 0.64  0.50  0.40 |

**Table S2 The sample amounts of 16S rRNA sequencing and SCFAs analysis among the L-VA and H-VA groups**

|  | **L-VA**  **(before eradication)** | **L-VA**  **(after eradication)** | **L-VA**  **(confirmation)** | **H-VA**  **(before eradication)** | **H-VA**  **(after eradication)** | **H-VA**  **(confirmation)** |
| --- | --- | --- | --- | --- | --- | --- |
| Sample number of  16s rRNA sequencing  Sample number of  SCFAs analysis | 30  19 | 28  18 | 24  17 | 23  14 | 23  14 | 22  12 |

L-VA: Dual therapy consisting of low dose of amoxicillin (1000mg bid) and VPZ (20mg bid); H-VA: Dual therapy consisting of high dose of amoxicillin (1000mg tid) and VPZ (20mg bid)

**Table S3 Topological indices among the *H. pylori*-positive group and the negative group**

| **Topological indexes**  **(empirical_network)** | ***H. pylori*-positive** | ***H. pylori*-negative** |
| --- | --- | --- |
| RMT_threshold  Average_nearest_neighbor_degree  Average_path_length  Betweenness_centrality  Closeness_centrality  Degree_assortativity  Degree_centralization  Density  Cluster_num  Diameter  Transitivity  Num_vertice  Num_edge  Modularity | 0.5  6.11  2.37  3401.53  0.98  0.43  201  0.15  2  3.02  0.44  35  92  0.50 | 0.5  12.30  1.83  2445.35  5.37  -0.13  370  0.28  1  2.63  0.39  35  165  0.40 |

**Table S4 Quantification of SCFAs in the *H. pylori*-positive group compared with the negative group**

| **SCFAs** | ***H. pylori*-positive**  **(median with first and third quartile)** | ***H. pylori*-negative**  **(median with first and third quartile)** | ***P* value** |
| --- | --- | --- | --- |
| Acetic acid  Propionic acid  Butyric acid  Isobutyric acid  Isovaleric acid  Valeric acid  Hexanoic acid  Total | 10390 (2609, 16736)  4535 (953, 10270)  4082 (1216, 7559)  193 (82, 463)  203 (88, 557)  474 (257,1162)  27 (16, 44)  20172 (6025, 38495) | 4470 (1828, 13665)  2694 (900, 5527)  3113 (954, 6566)  182 (70, 375)  249 (77, 343)  538 (85, 840)  18 (9, 39)  11877 (4249, 27095) | 0.26  0.23  0.66  0.66  0.68  0.46  0.15  0.30 |

SCFAs: Short-chain fatty acids.

**Table S5 Topological indices among the L-VA therapy groups**

| **Topological indexes**  **(empirical_network)** | **Before eradication** | **After eradication** | **Recheck** |
| --- | --- | --- | --- |
| RMT_threshold  Average_nearest_neighbor_degree  Average_path_length  Betweenness_centrality  Closeness_centrality  Degree_assortativity  Degree_centralization  Density  Cluster_num  Diameter  Transitivity  Num_vertice  Num_edge  Modularity | 0.5  11.04  1.78  860.99  1.36  0.33  227  0.33  2  2.39  0.68  29  133  0.32 | 0.5  7.17  2.16  1335.01  0.85  0.23  202  0.18  3  2.84  0.58  29  73  0.55 | 0.5  13.24  2.08  2470  5.35  0.22  276  0.30  1  3.33  0.73  32  150  0.38 |

**Table S6 Topological indices among the H-VA therapy groups**

| **Topological indexes**  **(empirical_network)** | **Before eradication** | **After eradication** | **Recheck** |
| --- | --- | --- | --- |
| RMT_threshold  Average_nearest_neighbor_degree  Average_path_length  Betweenness_centrality  Closeness_centrality  Degree_assortativity  Degree_centralization  Density  Cluster_num  Diameter  Transitivity  Num_vertice  Num_edge  Modularity | 0.5  11.13  2.20  6064.17  7.73  -0.13  512  0.17  1  3.03  0.36  38  124  0.50 | 0.5  9.86  2.38  4894.14  5.16  0.07  400  0.20  1  3.29  0.52  36  124  0.51 | 0.5  14.00  2.02  4151.04  7.05  -0.05  532  0.24  1  2.91  0.55  38  171  0.39 |

**Table S7 The quantification of SCFAs at the three time points of L-VA therapy**

| **SCFAs** | **L-1**  **(median with first and third quartile)** | **L-2**  **(median with first and third quartile)** | **L-3**  **(median with first and third quartile)** | ***P* value** |
| --- | --- | --- | --- | --- |
| Acetic acid  Propionic acid  Butyric acid  Isobutyric acid  Isovaleric acid  Valeric acid  Hexanoic acid  Total | 16167 (4827, 21529)  8430 (2378, 14914)  6283 (1360, 9139)  195 (85, 1111)  223 (103, 1057)  857 (274, 2386)  28 (17, 50)  32107 (13695, 55314) | 5847 (2186, 9431)  5756 (881, 9007)  3059 (755, 5030)  227 (77, 808)  218 (87, 704)  624 (64, 1133)  17 (7, 35)  17861 (3880, 27406) | 9991 (1807, 20315)  5701 (1002, 11952)  4023 (801, 8112)  360 (55, 595)  321 (50, 603)  667 (101, 1191)  21 (9, 35)  22922 (4279, 42010) | 0.068; L-1 vs. 2 0.013; L-2 vs. 3 0.318; L-1 vs. 3 0.379  0.394; L-1 vs. 2 0.169; L-2 vs 3 0.568; L1 vs. 3 0.531  0.193; L-1 vs. 2 0.066;  L-2 vs. 3 0.335; L-1 vs. 3 0.531  0.904; L-1 vs. 2 0.641; L-2 vs. 3 1.000; L-1 vs. 3 0.802  0.786; L-1 vs. 2 0.480; L-2 vs. 3 0.961; L-1 vs. 3 0.684  0.485; L-1 vs. 2 0.245; L-2 vs. 3 0.909; L-1 vs. 3 0.415  0.273; L-1 vs. 2 0.111; L-2 vs. 3 0.660; L-1 vs. 3 0.330  0.162; L-1 vs 2 0.042; L-2 vs 3 0.409; L-1 vs 3 0.471 |

SCFAs: Short-chain fatty acids. L-1: the timepoint of before eradication among L-VA group; L-2: the timepoint of after eradication among L-VA group; L-3: the timepoint of recheck among L-VA group.

**Table S8 The quantification of SCFAs at the three time points of H-VA therapy**

| **SCFAs** | **H-1**  **(median with first and third quartile)** | **H-2**  **(median with first and third quartile)** | **H-3**  **(median with first and third quartile)** | ***P* value** |
| --- | --- | --- | --- | --- |
| Acetic acid  Propionic acid  Butyric acid  Isobutyric acid  Isovaleric acid  Valeric acid  Hexanoic acid  Total | 8264 (2502,14749)  3585 (953, 11274)  2331 (842, 6398)  282 (84, 554)  325 (83, 567)  474 (196, 579)  27 (13, 44)  18023 (5982, 36892) | 2800 (1828, 4725)  2128 (1118, 2525)  1430 (572,2285)  139 (62, 261)  172 (80, 246)  196 (86, 351)  15 (9, 30)  7225 (3993, 10054) | 10012 (3935, 12472)  3663 (2414,6129)  2374 (1046, 4212)  185 (67, 392)  145 (78, 336)  176 (144, 647)  32 (17, 58)  18233 (8747, 25287) | 0.087; H-1 vs. 2 0.108; H-2 vs. 3 0.031; H-1 vs. 3 0.860  0.161; H-1 vs. 2 0.265; H-2 vs. 3 0.036; H-1 vs. 3 0.980  0.139; H-1 vs. 2 0.069; H-2 vs. 3 0.123; H-1 vs. 3 0.980  0.307; H-1 vs. 2 0.164; H-2 vs. 3 0.560; H-1 vs. 3 0.322  0.347; H-1 vs. 2 0.227; H-2 vs. 3 0.820; H-1 vs. 3 0.231  0.110; H-1 vs. 2 0.031; H-2 vs. 3 0.347; H-1 vs. 3 0.403  0.146; H-1 vs. 2 0.194; H-2 vs. 3 0.060; H-1 vs. 3 0.595  0.099; H-1 vs. 2 0.137; H-2 vs. 3 0.031; H-1 vs. 3 0.940 |

SCFAs: Short-chain fatty acids. H-1: the timepoint of before eradication among H-VA group; H-2: the timepoint of after eradication among H-VA group; H-3: the timepoint of recheck among H-VA group.
